# Postoperative Adjuvant Imatinib Therapy-Associated Nomogram to Predict Overall Survival of Gastrointestinal Stromal Tumor

**DOI:** 10.3389/fmed.2022.777181

**Published:** 2022-03-10

**Authors:** Xuechao Liu, Enyu Lin, Yuqi Sun, Xiaodong Liu, Zequn Li, Xuelong Jiao, Yi Li, Dong Guo, Peng Zhang, Xingyu Feng, Tao Chen, Zhaojian Niu, Zhiwei Zhou, Haibo Qiu, Yanbing Zhou

**Affiliations:** ^1^Department of General Surgery, Affiliated Hospital of Qingdao University, Qingdao, China; ^2^Department of Urology, Guangdong Provincial People's Hospital, Guangdong Academy of Medical Sciences, Guangzhou, China; ^3^Shantou University Medical College, Shantou, China; ^4^Department of General Surgery, Union Hospital, Tongji Medical College, Huazhong University of Science and Technology, Wuhan, China; ^5^Department of General Surgery, Guangdong General Hospital, Guangzhou, China; ^6^Department of General Surgery, Southern Medical University Nanfang Hospital, Guangzhou, China; ^7^Department of Gastric Surgery, Sun Yat-sen University Cancer Center, Guangzhou, China; ^8^State Key Laboratory of Oncology in South China, Collaborative Innovation Center for Cancer Medicine, Sun Yat-sen University Cancer Center, Guangzhou, China

**Keywords:** gastrointestinal stromal tumor, imatinib, diagnostic delay, prognosis, nomogram

## Abstract

**Background:**

Adjuvant imatinib therapy has been shown to improve overall survival (OS) of gastrointestinal stromal tumor (GIST) significantly. Few nomograms combining the use of adjuvant imatinib and clinicopathological characteristics estimate the outcome of patients. We aimed to establish a more comprehensive nomogram for predicting OS in patients with GIST.

**Methods:**

In total, 1310 GIST patients undergoing curative resection at four high-volume medical centers between 2001 and 2015 were enrolled. Independent prognostic factors were identified by multivariate Cox analysis. Eligible patients were randomly assigned in a ratio of 7:3 into a training set (916 cases) and a validation set (394 cases). A nomogram was established by R software and its predictive power compared with that of the modified National Institutes of Health (NIH) classification using time-dependent receiver operating characteristic (ROC) curves and calibration plot.

**Results:**

Age, tumor site, tumor size, mitotic index, postoperative imatinib and diagnostic delay were identified as independent prognostic parameters and used to construct a nomogram. Of note, diagnostic delay was for the first time included in a prognostic model for GIST. The calibrated nomogram resulted in predicted survival rates consistent with observed ones. And the decision curve analysis suggested that the nomogram prognostic model was clinically useful. Furthermore, time-dependent ROC curves showed the nomogram exhibited greater discrimination power than the modified NIH classification in 3- and 5-year survival predictions for both training and validation sets (all *P* < 0.05).

**Conclusions:**

Postoperative adjuvant imatinib therapy improved the survival of GIST patients. We developed and validated a more comprehensive prognostic nomogram for GIST patients, and it could have important clinical utility in improving individualized predictions of survival risks and treatment decision-making.

## Introduction

Gastrointestinal stromal tumors (GISTs) are the most common mesenchymal neoplasms originating in the gastrointestinal tract. GISTs are characterized by aberrant expression of the receptor tyrosine kinase KIT, which is detectable in approximately 95% cases (1, 2). The most common primary site of GIST is stomach (60%), followed by small intestine (30%), colorectum (10%), and esophagus (5%). Rarely, the tumors occur in the mesentery, omentum, pelvis, and retroperitoneum (so-called extragastrointestinal GIST). Constitutively activating mutations of KIT or platelet-derived growth factor receptor alpha (PDGFRA) play a key role in the biology of GISTs, thereby providing a rationale for molecularly targeted therapy (3–5). Indeed, inhibition of KIT and PDGFRA with imatinib (Glivec, Novartis) has yielded remarkable improvements in long-term outcomes (6–8). Unfortunately, the high recurrence rate remains an unsettling problem. Therefore, clinicians continue to seek independent prognostic factors to build a more comprehensive and accurate model to improve disease management (9).

Among existing risk-stratification tools, the most widely used are the modified National Institutes of Health (mNIH) classification and the Armed Forces Institute of Pathology (AFIP) myriad (10, 11). These systems incorporate well-established independent risk factors including primary tumor size, mitotic index, tumor site, and tumor rupture. Of note, other clinicopathological characteristics such as age, sex, histological subtype and postoperative therapy can also influence patient outcomes (12, 13). Adjuvant imatinib treatment has been shown to improve overall survival (OS) significantly. Furthermore, in the past years the potential value of diagnostic delay as a prognostic factor has been explored in many other malignancies. Elfgen et al. (14) reported that diagnostic delay led to tumor growth and/or tumor stage upgrade in 8.9% of basal-like breast cancer, which had the lowest overall survivals. However, Salvador et al. (15) found short diagnostic intervals were significantly associated with higher 5-year mortality in rectal but not in colon cancers, and diagnostic delay seemed not to be associated with poorer outcome. However, in GIST, the prognostic value of diagnostic delay currently remains unclear. To facilitate clinical practice and decision-making, several statistical prediction models integrating diverse prognostic factors have been established to identify homogeneous high-risk patient groups (16–18). Among these decision-making tools, nomogram has been shown in some malignancies to be superior in predicting clinical outcomes (19, 20). By integrating several continuous variables, a nomogram can accurately quantitatively predict the probability of a particular outcome with a single metric.

In this study, we present a novel prognostic nomogram for patients undergoing curative resection for GIST. Independent prognostic factors were identified from a large-scale multicenter retrospective analysis. Based on these factors, we then constructed a nomogram, compared its predictive power with the mNIH system, and further assessed it accuracy by comparing its survival outcome predictions with actual observations.

## Materials and Methods

### Study Population

Our study retrospectively analyzed 1,310 patients undergoing curative resection for GIST between 2001 and 2015 at four medical centers in China, including Sun Yat-sen University Cancer Center, The Union Hospital Huazhong University of Science and Technology, Guangdong General Hospital and Southern Medical University Nanfang Hospital. All are high-capacity centers located in areas of high GIST incidence in China. Two investigators from each center approved the final dataset before it was pooled. After reviewing all deidentified data, reports were made to solve data inconsistencies by personal correspondence. All enrolled patients were diagnosed with GISTs based on postoperative histological specimen according to standard guidelines. Clinical, pathological and survival data were recorded in all cases, including age, sex, histological subtype, performance status, diagnostic delay, postoperative tumor characteristics, postoperative imatinib therapy and survival duration. The modified mNIH criteria, the most commonly used staging system for GISTs, were used for risk stratification. moderate- and high-risk patients routinely received adjuvant imatinib therapy after surgery as per standard guidelines.

Patient inclusion criteria were as follows: (1) the patient had localized primary GIST for which he/she underwent curative surgery, (2) the patient did not present with distant metastases at diagnosis, (3) there was no other synchronous malignancy, (4) the patient had not received preoperative treatment with imatinib or other tyrosine kinase inhibitor, and (5) complete clinicopathological and follow-up data were available for the patient. Furthermore, we excluded individuals who died within 1 month after surgery, upon review all of whom were found to have died of severe postoperative complications including anastomotic leakage, organ infection, bleeding, and multiple organ dysfunction syndrome (MODS). Finally, 1,310 GIST patients were enrolled in our study.

### Patient Follow-Up

A strict postoperative monitoring program was conducted annually for very low- or low-risk patients and every 6 months for intermediate- or high-risk patients. Follow-up assessment comprised medical history, physical examination, blood test, endoscopy, and dynamic abdominal pelvic computerized tomography scan. Side effects of adjuvant treatment and postoperative recovery were also assessed at each follow-up. OS was defined as the time interval from the date of surgery to death or last follow-up. The final follow-up date for the study was February 2016 and the median follow-up period was 36.6 (range: 2–174) months.

### Ethics Statement

The study was approved by the research ethics board of the Affiliated Hospital of Qingdao University (ethics approval no. QDFY WZLL26688) and complied with the standards of the Declaration of Helsinki. Written informed consent was obtained from either the patient or the patient's family.

## Statistical Analysis

Results were presented as mean with 95% confidence intervals (CI). Statistical methods included Kaplan–Meier method for the overall cumulative survival rate, the log-rank test for statistical differences among groups, and the Cox proportional hazards model for multivariate analysis using a stepwise procedure. If a continuous variable met the assumption of linearity in the logit regression analysis, it was then categorized by the optimal cutoff value as determined by receiver operating characteristics (ROC) curve analysis. Variables were assessed for co-linearity with the linear regression model. A nomogram was generated by the R package rms to predict the probabilities of 3- and 5-year OS. Time-dependent ROC curves were constructed to assess the predictive accuracy of prognostic models, and area under the ROC curve (AUC) was calculated to compare the discriminatory ability. Decision curve analysis (DCA) were used to evaluate the clinical benefit of the nomogram model by quantifying net benefits at different threshold probabilities. The curves of treat-all-patients scheme (the highest net benefit) and the treat-none scheme (no net benefit) were set as two references (21, 22). Calibration plots were performed by comparing the predicted probability of OS with the observed outcome, in which the 45-degree line was used as the optimal model. A *P*-value < 0.05 was considered statistically significant. All statistical analyses were conducted using R software version 3.6.1 (https://www.r-project.org/) and SPSS version 19.0 (IBM Corporation, Armonk, NY, USA).

## Results

### Patient's Characteristics

The clinicopathological characteristics of patients, surgeons, and hospitals are listed in Table 1. Of the 1,310 enrolled patients (684 men), median (range) age at the time of diagnosis was 59 (20 to 91) years. Most tumors were located in the stomach (65.6%) and the median tumor size was 4.5 cm (range 0.1–45.0). Based on the modified NIH classification (mNIH), 213 (16.3%) patients were classified as very low risk, 414 (31.6%) low risk, 195 (14.9%) intermediate risk, and 488 (37.3%) high risk.

**Table 1:** Clinicopathologic characteristics of patients with gastrointestinal stromal tumors.

	**Total (%)**	**Training set**	**Validation set**	***P*-value**
Age (years)				0.295
<60	706 (53.9%)	485 (52.9%)	221 (56.1%)	
≥60	604 (46.1%)	431 (47.1%)	173 (43.9%)	
Sex				0.835
Male	684 (52.2%)	480 (52.4%)	204 (51.8%)	
Female	626 (47.8%)	436 (47.6%)	190 (48.2%)	
Tumor size (cm)				0.316
≤ 5	754 (57.6%)	519 (56.7%)	235 (59.6%)	
>5	556 (42.4%)	397 (43.3%)	159 (40.4%)	
Mitotic index (/50 HPF)				0.025
≤ 5	949 (72.4%)	647 (70.6%)	302 (76.6%)	
>5	361 (27.6%)	269 (29.4%)	92 (23.4%)	
Tumor site				0.117
Stomach	859 (65.6%)	613 (66.9%)	246 (62.4%)	
Non-stomach	451 (34.4%)	303 (33.1%)	148 (37.6%)	
Histological subtype				0.888
Spindle type	1,178 (89.9%)	823 (89.8%)	355 (90.1%)	
Epithelioid/mixed type	132 (10.1%)	93 (10.2%)	39 (9.9%)	
Tumor rupture				0.016
No	1,301 (99.3%)	913 (99.7%)	388 (98.5%)	
Yes	9 (0.7%)	3 (0.3%)	6 (1.5%)	
Performance status				0.718
0	655 (50.0%)	455 (49.7%)	200 (50.8%)	
≥1	655 (50.0%)	461 (50.3%)	194 (49.2%)	
Diagnostic delay				0.667
No	793 (60.5%)	551 (60.2%)	242 (61.4%)	
Yes	517 (39.5%)	365 (39.8%)	152 (38.6%)	
Postoperative imatinib				0.285
No	1,009 (77.0%)	713 (77.8%)	296 (75.1%)	
Yes	301 (23.0%)	203 (22.2%)	98 (24.9%)	

### The Proposal of Diagnostic Delay

Diagnostic delay, defined as time to diagnosis exceeding 60 days by receiver operating characteristics (ROC) curve analysis, was present in 517 (39.5%) patients. Moreover, presence of diagnostic delay was significantly associated with poorer performance status and a lower risk classification (*P* < 0.001 for both associations; Table 2). A related metric symptom-to-diagnosis interval (SDI) was calculated as the time from the onset of tumor symptoms to the diagnosis of GIST. We found that a shorter SDI was associated with vomiting and gastrointestinal bleeding, and longer one with abdominal pain and abdominal mass (all *P* < 0.05). Among the four risk groups, the median SDI was significantly longer in very low-risk patients than in other groups (all adjusted *P* < 0.001).

**Table 2:** Relationships between diagnostic delay with clinicopathological features.

	**Timely diagnosis**	**Diagnostic delay**	***P*-value**
	**(*****n*** **=** **793)**	**(*****n*** **=** **517)**	
Sex			0.369
Male	422	262	
Female	371	255	
Age (years)			0.966
<60	427	279	
≥60	366	238	
Tumor size (cm)			0.335
≤ 5	448	306	
>5	345	211	
Mitotic index (/50 HPF)			0.572
≤ 5	570	379	
>5	223	138	
Tumor site			0.906
Stomach	519	340	
Non-stomach	274	177	
Histological subtype			0.561
Spindle type	710	468	
Epithelioid/mixed type	83	49	
Tumor rupture			0.322
No	789	512	
Yes	4	5	
Performance status			<0.001
0	431	224	
≥1	362	293	
Modified NIH classification			<0.001
Very low	93	120	
Low	277	137	
Intermediate	130	65	
High	293	195	

### Nomogram Variable Screening

**Figure 1:**
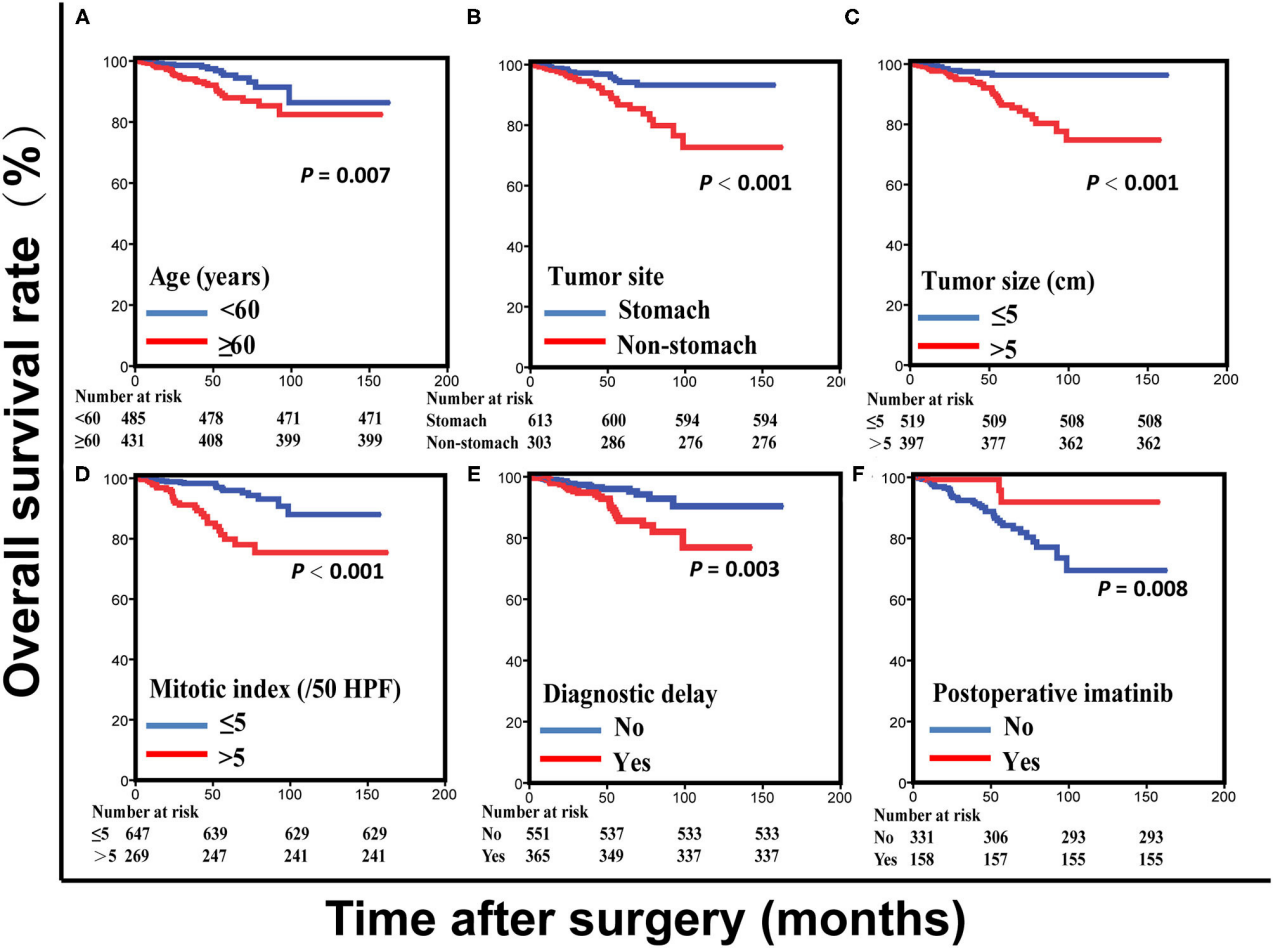
Overall survival curves for patients with gastrointestinal stromal tumors based on age **(A)**, tumor site **(B)**, tumor size **(C)**, mitotic index **(D)**, and diagnostic delay **(E)** in the training set. Overall survival curves for intermediate- and high-risk patients with gastrointestinal stromal tumors based on postoperative imatinib **(F)** in the training set.

Results from the multivariate analysis in all patients indicated that age, tumor site, tumor size, mitotic index, postoperative imatinib and diagnostic delay were all significantly associated with OS (all *P* < 0.05; Supplementary Table 1). Notably, diagnostic delay maintained prognostic significance when patients were stratified by tumor site and performance status (all *P* < 0.05). Subsequently, to build a prognosis model, patients were randomly assigned in a ratio of 7:3 into a training set (916 cases) and a validation set (394 cases). Results of the univariate analysis in the training set were showed in Table 3. Of note, there was a positive correlation between postoperative imatinib and mNIH classification, while there was a negative correlation between mNIH classification and OS. Therefore, we included -postoperative imatinib on multivariate analysis and found that age, tumor site, tumor size, mitotic index, postoperative imatinib and diagnostic delay were all significantly associated with OS (all *P* < 0.05; Figure 1). Furthermore, we drew similar conclusion when including all the ten variables on the multivariate Cox analysis (data not shown). As the repeated identifications of diagnostic delay as an independent factor strongly suggests its potential merit in survival prediction, it was used along with other five clinical attributes in the subsequent nomogram construction and survival prediction.

**Table 3:** Univariate and multivariate analyses of patients with gastrointestinal stromal tumors in the training set.

	**Univariate analysis**	**Multivariate analysis**
	**HR (95 % CI)** ***P*****-value**	**HR (95 % CI)** ***P*****-value**
Sex	0.558	
Male	1.00	
Female	1.189 (0.667, 2.121)	
Age (years)	0.009	0.003
<60	1.00	1.00
≥60	2.322 (1.239, 4.354)	2.609 (1.382, 4.926)
Tumor size (cm)	<0.001	0.047
≤ 5	1.00	1.00
>5	3.723 (1.889, 7.338)	2.121 (1.011, 4.449)
Mitotic index (/50 HPF)	<0.001	<0.001
≤ 5	1.00	1.00
>5	4.497 (2.486, 8.135)	3.914 (2.083, 7.357)
Tumor site	0.001	0.004
Stomach	1.00	1.00
Non-stomach	2.726 (1.515, 4.907)	2.442 (1.327, 4.496)
Histological subtype	0.054	
Spindle type	1.00	
Epithelioid/mixed type	1.995 (0.990, 4.023)	
Tumor rupture	0.748	
No	1.00	
Yes	—	
Performance status	0.510	
0	1.00	
≥1	1.215 (0.681, 2.170)	
Diagnostic delay	0.004	0.002
No	1.00	1.00
Yes	2.389 (1.321, 4.320)	2.566 (1.413, 4.659)
Postoperative imatinib	0.146	0.025
No	1.00	1.00
Yes	0.467 (0.167, 1.304)	0.304 (0.108, 0.860)

### Development and Validation of Nomograms

**Figure 2:**
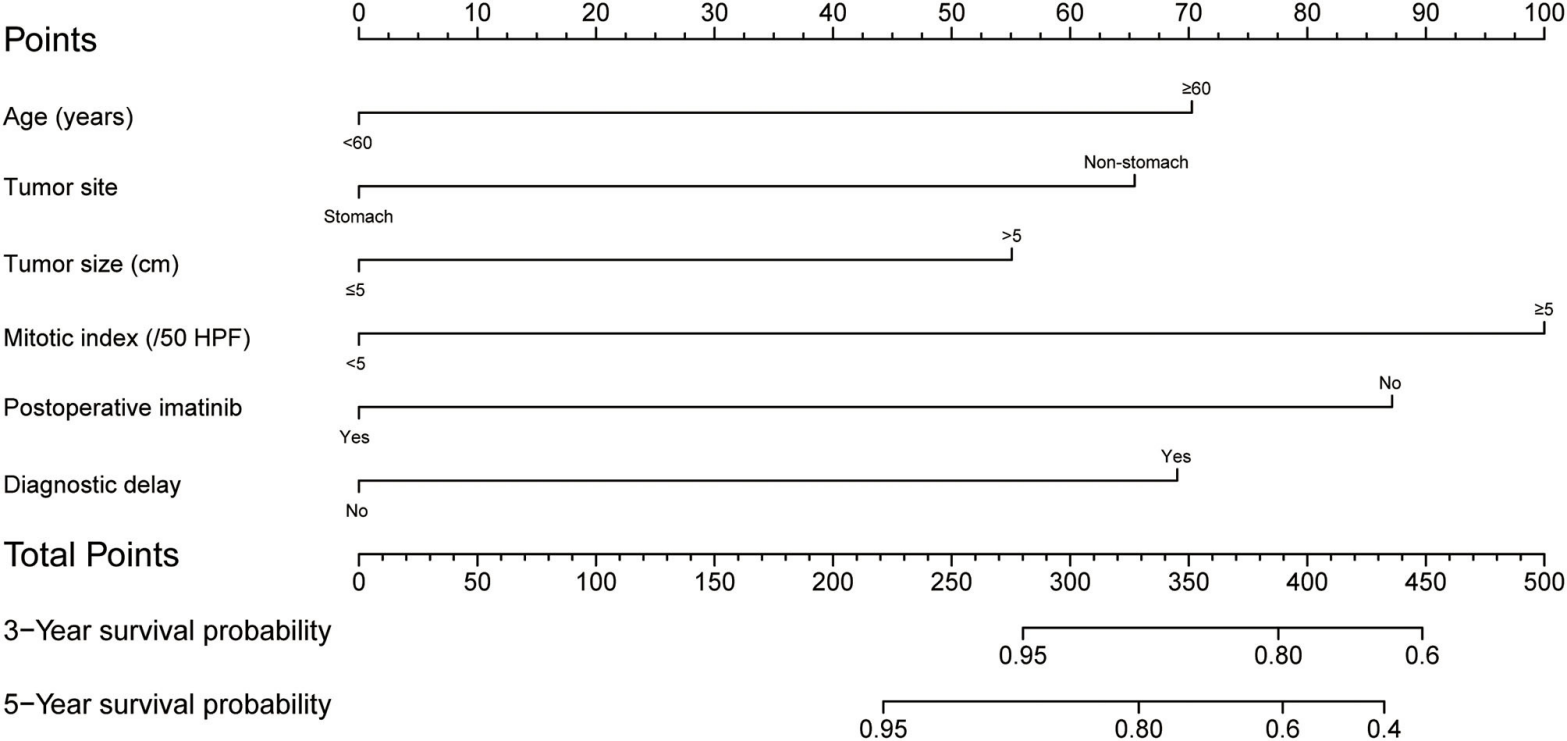
Postoperative nomogram for predicting 3- and 5-year overall survival in patients undergoing curative resection for gastrointestinal stromal tumor.

A prognostic nomogram yields quantitative probabilities of survival at certain time points, for which a higher total score indicates a worse clinical outcome. Figure 2 illustrates the prognostic nomogram established for 3- and 5-year OS based on the six prognostic factors identified with the training set. The C-index value was 0.812 [95% CI = 0.749-0.875] in the training set and 0.849 (95% CI = 0.769–0.929) in the validation set.

**Figure 3:**
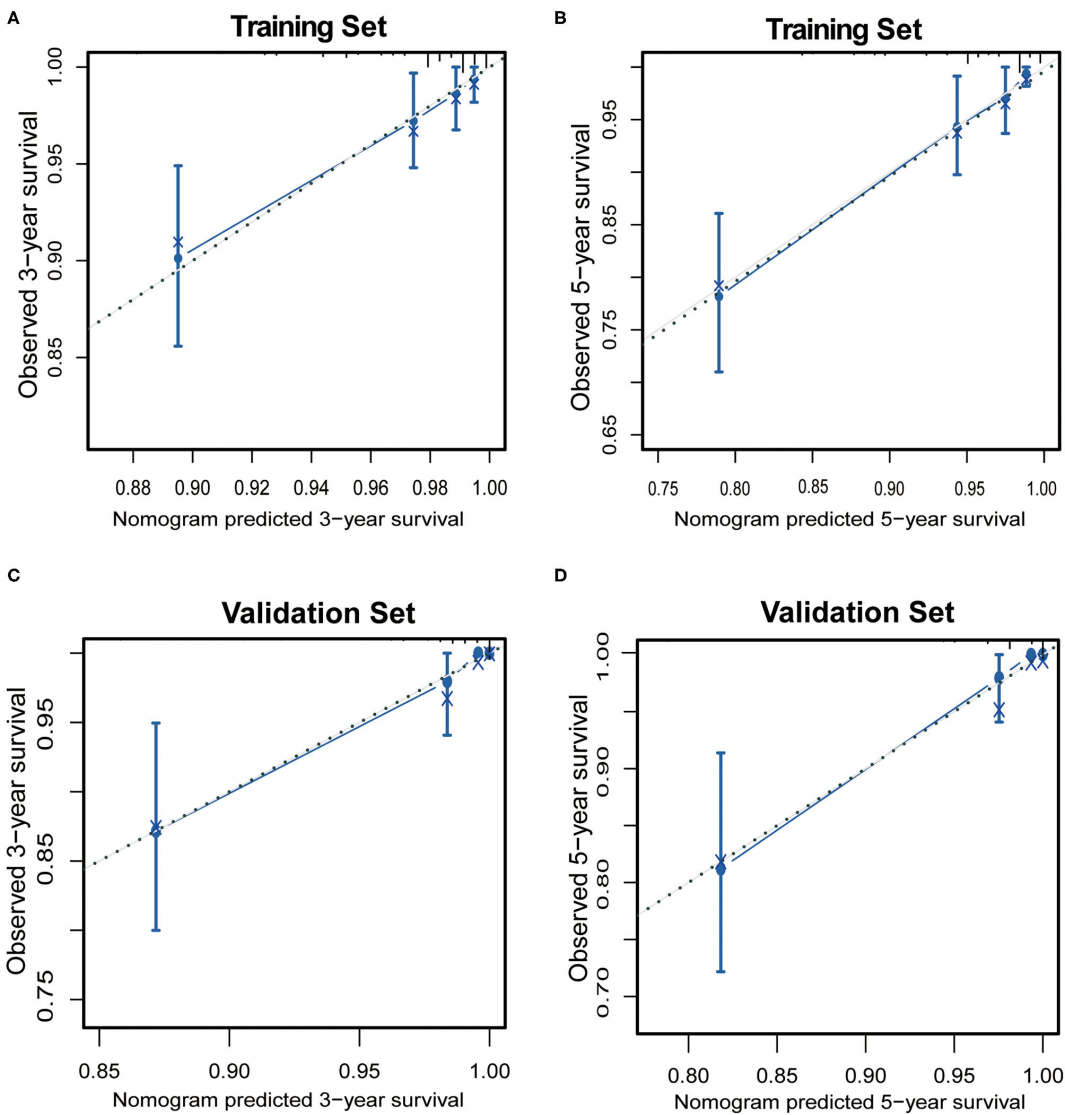
The calibration of the nomograms for 3-year and 5-year overall survival in the training set **(A,B)** and the validation set **(C,D)**. The x-axis represents the nomogram-predicted survival rate, and the y-axis represents actual survival rate and 95% confidence intervals. The dotted line represents the ideal correlation between predicted and actual survival rate.

For performance evaluation of this nomogram, calibration curves were generated with the bootstrap approach to assess the concordance between the predicted and the actual outcomes. As shown in Figure 3, calibration plots of the nomogram predicting 3- and 5-year OS performed well with the ideal model in the training and validation set.

**Figure 4:**
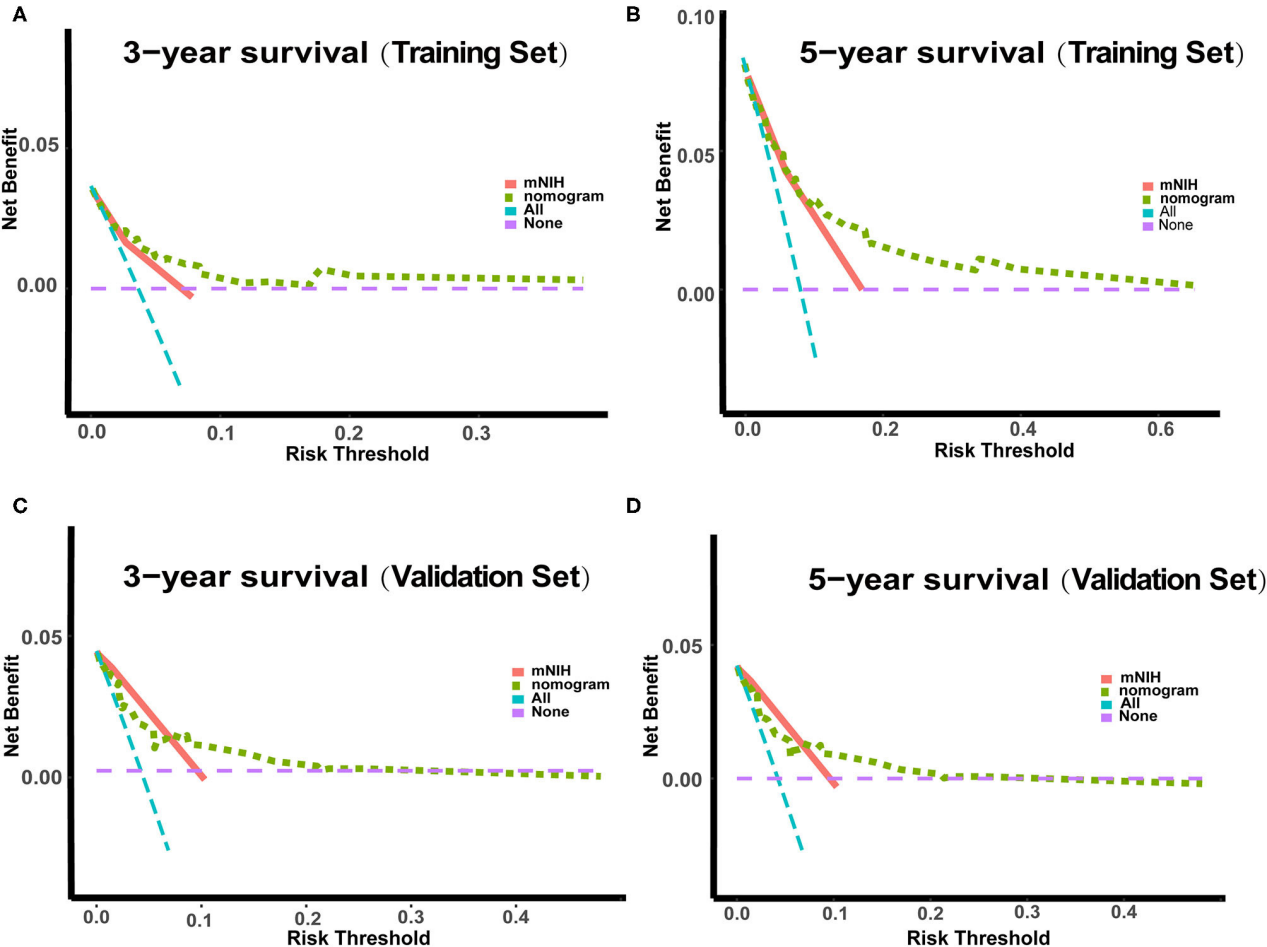
Decision curve analysis (DCA) of the nomogram and the modified NIH risk classification. The y-axis represents the net benefit, whereas the x-axis represents the threshold probability. **(A)** 3-year survival benefit in the training set. **(B)** 5-year survival benefit in the training set. **(C)** 3-year survival benefit in the validation set. **(D)** 5-year survival benefit in the validation set.

In the DCA, the nomogram could better predict 3- and 5-year OS, as it added more net benefits compared with the widely accepted mNIH classification for almost all threshold probabilities in both the training and validation sets (Figure 4). Therefore, the DCA analysis demonstrated that our prognostic nomogram had better predictive capability and accuracy.

### Predictive Ability of the Nomogram Compared With the MNIH Classification

**Figure 5:**
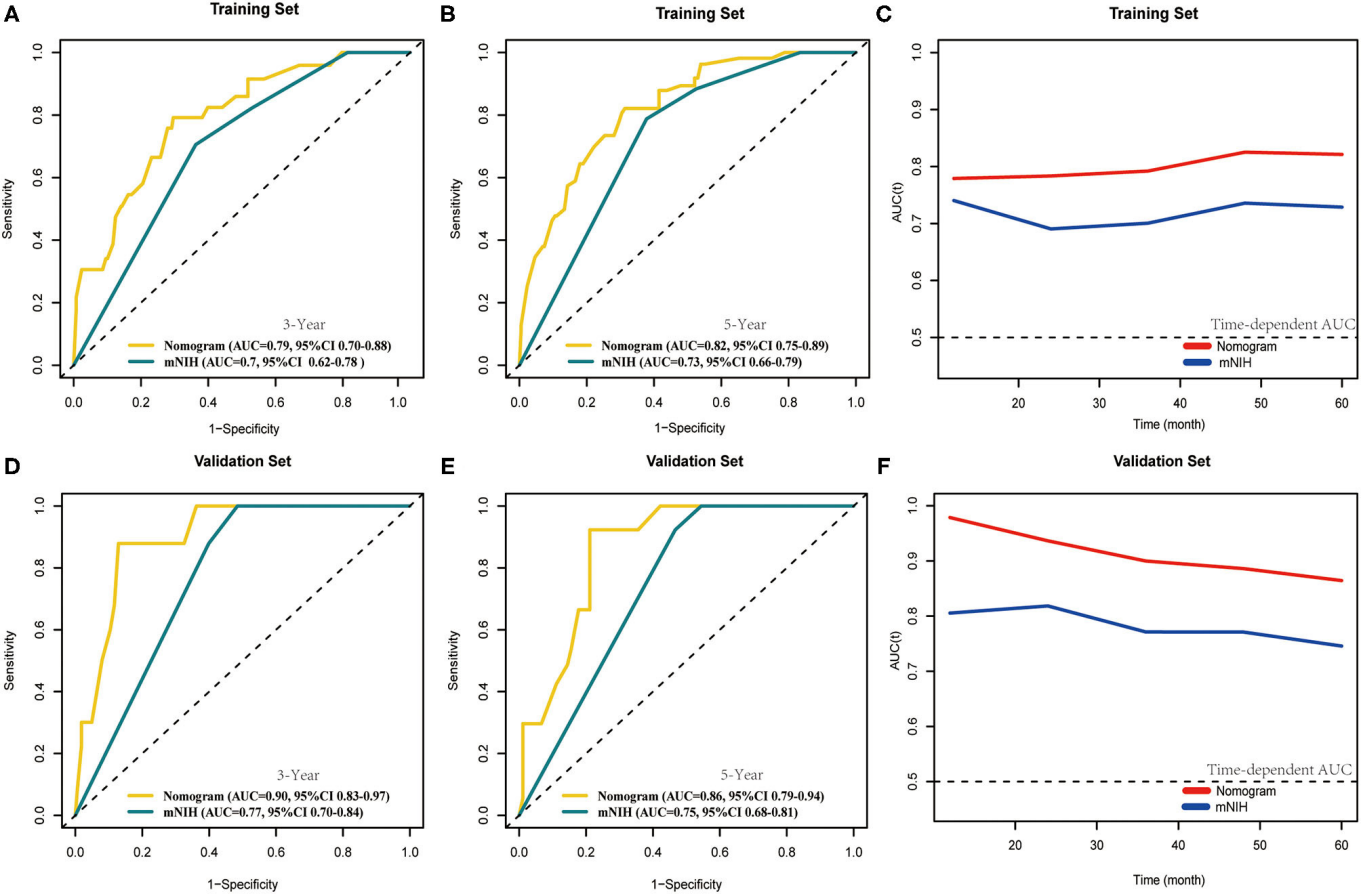
Comparison of the area under curves (AUCs) of the time-dependent receiver operating characteristic (tROC) analysis of our nomogram and the modified NIH risk classification. tROC curves were plotted to evaluate the performance of the two prognostic models in predicting 3-year and 5-year overall survival in the training set **(A,B)** and the validation set **(D,E)** and AUC of these curves were calculated for comparison. tROC curves of the nomogram for 3- and 5-year overall survival prediction in the training set **(C)** and the validation set **(F)**.

Furthermore, we compared the discrimination ability of the nomogram with that of the widely accepted mNIH (Figure 5). In the training set, the AUCs of our nomogram predicting 3- and 5-year OS were 0.79 and 0.82, respectively, whereas the corresponding AUCs of mNIH were 0.70 and 0.73. Moreover, AUCs of 3- and 5-year OS predictions were also significantly higher with the nomogram (AUCs 0.90 and 0.86) than with mNIH (0.77 and 0.75) in the validation set (*P* < 0.05 for all comparisons). Therefore, time-dependent ROC analysis of both training and validation sets demonstrated a greater discriminatory power in 3- and 5-year survival prediction our nomogram has compared with mNIH. Taken together, these results suggest superiority of our prognostic nomogram over mNIH in predictive ability for patients with operable GIST.

## Discussion

The success of targeted therapy with selective tyrosine kinase inhibitor (TKI) in treating GIST has generated considerable interest in this field in recent years (23). In particular, there has been a continuing effort to identify independent prognostic factors for improved risk stratification, as accurate risk stratification after surgery helps determine adjuvant therapy plans and the intensity of postoperative surveillance (24–26). In the past decades, various risk stratification systems for patients with GIST have been proposed. In 2002, Fletcher et al. (27) developed a consensus approach. Based on long-term follow-up results, Miettinen et al. (11) put forward the AFIP criteria using tumor size, mitosis count and tumor site as independent risk factors. The NIH system was subsequently modified, adding a new factor, tumor rupture, and has since been the most widely used tool to identify high-risk groups (10). More recently, other schemes began to emerge for estimating individualized outcomes, including the prognostic contour maps and the Memorial Sloan Kettering Cancer Center (MSKCC) nomogram (16, 28). However, current classification schemes are still unable to explain all the biological behaviors and outcome variations. Therefore, it is a focus in clinical research to create a more effective prognosis model (29, 30).

Our study presents a prognostic nomogram with improved accuracy and specificity based on clinicopathological characteristics and adjuvant therapy for GIST.

A nomogram model was chosen because its ability to extend the standard stratification tools on an individualized basis by integrating tumor- and patient-related factors. Multivariate analyses were first conducted and identified age, tumor site, tumor size, mitotic index, postoperative imatinib, and diagnostic delay as independent prognostic factors. In nomogram construction, we included age, diagnostic delay and postoperative imatinib therapy in addition to well-established factors such as tumor size and mitotic index to comprehensively evaluate prognosis. Calibration plot analysis showed high concordance between predicted and actual survival rates. Moreover, in time-dependent ROC analysis our model exhibited greater discriminatory ability than the classic modified NIH classification. Therefore, inclusion of age, diagnostic delay, and postoperative imatinib therapy as independent risk factors conferred the nomogram model with additional predictive power. This may have resulted from a more comprehensive delineation of the underlying tumor biology and postoperative management enabled by including these additional independent risk factors.

In recent years, several nomograms have been created to guide the management of operable GIST according to the Surveillance, Epidemiology, and End Results (SEER) program database (31, 32). However, these studies did not take into account the aspect of adjuvant imatinib therapy, which would most likely affect the accuracy of risk stratification. According to current guidelines, adjuvant imatinib at 400 mg daily for ≥3 years is recommended based on the SSG XVIII/AIO trial, which demonstrated an improvement in both RFS and OS for high risk patients. Adjuvant imatinib is also given for 1 years after resection of intermediate risk tumors. Many patients finally gave up imatinib treatment due to huge expenses, especially ten years ago. Therefore, most study lacked associated data for a further analysis. In our study 1,310 GIST patients undergoing curative resection at four high-volume medical centers between 2001 and 2015 were enrolled, and only 301 (23.0%) patients received imatinib treatment. We found adjuvant imatinib therapy improved the survival of GIST patients and were used to construct a nomogram. Of the 488 high risk patients, 187 (38.3%) patients received postoperative imatinib more than 1 year. The median number of months treated in patients who tolerated treatment was 26.2 (range 13.5 to 99.1). Furthermore, some intermediate-risk patients were also not treated with a standard dose and duration of imatinib, which might have confounded our results. In our study, the most common adverse effects of adjuvant imatinib were fluid retention, rash, nausea and diarrhea. Rarely (<1%) severe adverse effects, such as liver toxicity or severe rash, might preclude the use of imatinib. Furthermore, mutation analysis is particularly important for decision-making regarding the use of adjuvant imatinib. Unfortunately, there was only a small number of patients with genetic mutation information acquired in our database.

Another interesting feature in the present study is the identification of diagnostic delay as an independent prognostic factor and its use in our novel nomogram. Relationship between diagnostic delay and clinical outcomes has been reported in many malignancies. However, up to now no definitive data exist to prove whether diagnostic delay has an impact on long-term prognosis in GIST. Our results suggest that diagnostic delay have potent prognostic value in GIST and constitute a promising complementation to current risk classification systems. By combining the well-established risk factors with the ones identified from 1,310 patients, we built a prognostic nomogram with greater accuracy and discriminatory power than the standard modified NIH classification. Due to the retrospective study design and data collection, missing data were inevitable. To maintain the representativeness of the study population, we kept as many cases as possible and used all available information for each analysis, which may have resulted in biased estimates. Finally, 1310 consecutive GIST patients were enrolled to build a prognosis model. Patients were randomly assigned in a ratio of 7:3 into a training set and a validation set by R software. Furthermore, although there exists the heterogeneity and sample selection bias, we think it is reasonable to conclude that they are difficult to alter the conclusions.

Our prognostic model may have important clinical value in predicting clinical outcomes and informing postoperative therapeutic intervention for individual patients. The model can also be used to facilitate risk communication between doctors and patients. Moreover, through providing evidence supporting early monitoring of the high-risk GIST patients, our model can help identify high-risk patients for prospective clinical trials (33). With these potential benefits in mind, we encourage prospective randomized controlled studies to further validate our nomogram. Of note, whether high-risk patients benefit from a prolonged imatinib treatment would also be of considerable interest in clinical practice.

Although the nomogram performed well, our study had several limitations. Patients with tumor rupture have a high risk of GIST recurrence. However, only 0.7% of the patients with tumor rupture were included in our studies and there was no correlation between tumor rupture and OS, which might affect the accuracy of our model. Moreover, our conclusions might be strengthened by the progression/recurrence-free survival analysis. Unfortunately, we lacked associated data to further investigate this idea. Finally, a small number of patients were not treated with a standard dose and duration of imatinib, which may have confounded the results. However, our model was based on a large-scale multicenter sample size whose statistical power was generally strong. More importantly, we constructed an adjuvant imatinib therapy-associated prognostic model to predict the outcome of GIST accurately and individually in the era of TKIs.

## Conclusions

In summary, based on a retrospective, multi-center analysis of 1,310 GIST patients undergoing curative resection, six independent prognostic factors were identified and used to construct a prognostic nomogram. Compared with the modified NIH criteria, our nomogram included additional factors such as diagnostic delay and postoperative treatment, which provided additional delineation of the disease course. In terms of performance, the nomogram model showed increased discriminatory ability in predicting 3- and 5-year overall survival probabilities compared with modified NIH classification and also predicted survival rates highly consistent with observed ones. Thus, our novel model could have important clinical utility in improving individualized predictions of survival risks and treatment decision-making.

We invite prospective randomized controlled studies to provide this nomogram model with further validation.

## Data Availability Statement

The raw data supporting the conclusions of this article will be made available by the authors, without undue reservation.

## Ethics Statement

The studies involving human participants were reviewed and approved by research Ethics Board of the Affiliated Hospital of Qingdao University. The patients/participants provided their written informed consent to participate in this study. Written informed consent was obtained from the individual(s) for the publication of any potentially identifiable images or data included in this article.

## Author Contributions

XuL, ZZ, HQ, and YZ: conceptualization. XuL, EL, YS, XiL, ZL, and ZN: data curation. EL, XJ, and YL: formal analysis. YS and YL: investigation. YS, XiL, and XJ: methodology. YZ: project administration. XiL, ZL, and YL: software. ZN, ZZ, and YZ: supervision. XJ: validation. ZL: visualization. XuL, EL, PZ, XF, and TC: writing—original draft. XF and TC: writing—review and editing. All authors have read and agree to publish the manuscript.

## Funding

This work was supported by the National Science Foundation of China (Grant No. 82002528).

## Conflict of Interest

The authors declare that the research was conducted in the absence of any commercial or financial relationships that could be construed as a potential conflict of interest.

## Publisher's Note

All claims expressed in this article are solely those of the authors and do not necessarily represent those of their affiliated organizations, or those of the publisher, the editors and the reviewers. Any product that may be evaluated in this article, or claim that may be made by its manufacturer, is not guaranteed or endorsed by the publisher.

## References

[1] Wu CE Tzen CY Wang SY. Clinical diagnosis of gastrointestinal stromal tumor (GIST): from the molecular genetic point of view. Cancers. (2019) 11:679. 10.3390/cancers11050679 31100836 PMC6563074

[2] Liegl-Atzwanger B Fletcher JA Fletcher CD. Gastrointestinal stromal tumors. Virchows Arch. (2010) 456:111–27. 10.1007/s00428-0891-y 20165865

[3] Serrano C George S. Gastrointestinal stromal tumor: challenges and opportunities for a new decade. Clin Cancer Res. (2020) 26:5078–85. 10.1158/1078-0432.CCR-20-1706 32601076

[4] Farag S Smith MJ Fotiadis N Constantinidou A Jones RL. Revolutions in treatment options in gastrointestinal stromal tumours (GISTs): the latest updates. Curr Treat Options Oncol. (2020) 21:55. 10.1007/s11864-020-00754-8 32462367 PMC7253383

[5] Lasota J Miettinen M. Clinical significance of oncogenic KIT and PDGFRA mutations in gastrointestinal stromal tumours. Histopathology. (2008) 53:245–66. 10.1111/j.1365-2559.2008.02977.x 18312355

[6] Joensuu H Wardelmann E Sihto H Eriksson M Sundby Hall K Reichardt A. Effect of KIT and PDGFRA mutations on survival in patients with gastrointestinal stromal tumors treated with adjuvant imatinib: an exploratory analysis of a randomized clinical trial. JAMA Oncol. (2017) 3:602–9. 10.1001/jamaoncol.2016.5751 28334365 PMC5470395

[7] Heinrich MC Rankin C Blanke CD Demetri GD Borden EC Ryan CW. Correlation of long-term results of imatinib in advanced gastrointestinal stromal tumors with next-generation sequencing results: analysis of phase 3 SWOG intergroup trial S0033. JAMA Oncol. (2017) 3:944–52. 10.1001/jamaoncol.2016.6728 28196207 PMC5727908

[8] Joensuu H Eriksson M Sundby Hall K Reichardt A Hartmann J T Pink D. Adjuvant imatinib for high-risk GI stromal tumor: analysis of a randomized trial. J Clin Oncol. (2016) 34:244–50. 10.1200/JCO.2015.62.9170 26527782

[9] Casali PG Zalcberg J Le Cesne A Reichardt P Blay JY Lindner LH. Ten-year progression-free and overall survival in patients with unresectable or metastatic GI stromal tumors: long-term analysis of the European organisation for research and treatment of cancer, Italian sarcoma group, and Australasian gastrointestinal trials group intergroup phase III randomized trial on imatinib at two dose levels. J Clin Oncol. (2017) 35:1713–20. 10.1200/JCO.2016.71.0228 28362562

[10] Joensuu H. Risk stratification of patients diagnosed with gastrointestinal stromal tumor. Hum Pathol. (2008) 39:1411–9. 10.1016/j.humpath.2008.06.025 18774375

[11] Miettinen M Lasota J. Gastrointestinal stromal tumors: pathology and prognosis at different sites. Semin Diagn Pathol. (2006) 23:70–83. 10.1053/j.semdp.2006.09.001 17193820

[12] Patel DJ Lutfi W Eguia E Sweigert P Knab L Abood G. Adjuvant systemic therapy for small bowel gastrointestinal stromal tumor (GIST): is there a survival benefit after R0 resection? Surgery. (2020) 168:695–700. 10.1016/j.surg.2020.04.069 32713755

[13] Ge XY Lei LW Ge F Jiang X. Analysis of risk factors of gastrointestinal stromal tumors in different age groups based on SEER database. Scand J Gastroenterol. (2019) 54:480–4. 10.1080/00365521.2019.1604798 31017491

[14] Elfgen C Varga Z Reeve K Moskovszky L Bjelic-Radisic V Tausch C. The impact of distinct triple-negative breast cancer subtypes on misdiagnosis and diagnostic delay. Breast Cancer Res Treat. (2019) 177:67–75. 10.1007/s10549-019-05298-6 31154578

[15] Pita-Fernández S González-Sáez L López-Calviño B Seoane-Pillado T Rodríguez-Camacho E Pazos-Sierra A. Effect of diagnostic delay on survival in patients with colorectal cancer: a retrospective cohort study. BMC Cancer. (2016) 16:664. 10.1186/s12885-016-2717-z 27549406 PMC4994409

[16] Joensuu H Vehtari A Riihimäki J Nishida T Steigen S E Brabec P. Risk of recurrence of gastrointestinal stromal tumour after surgery: an analysis of pooled population-based cohorts. Lancet Oncol. (2012) 13:265–74. 10.1016/S1470-2045(11)70299-6 22153892

[17] Wu J Zhang H Li L Hu M Chen L Xu B. A nomogram for predicting overall survival in patients with low-grade endometrial stromal sarcoma: a population-based analysis. Cancer Commun. (2020) 40:301–12. 10.1002/cac2.12067 32558385 PMC7365459

[18] Dai Y Qiang W Lin K Gui Y Lan X Wang D. An Immune-Related Gene Signature For Predicting Survival And Immunotherapy Efficacy In Hepatocellular Carcinoma. (2020). 10.21203/rs.3.rs-36927/v1 PMC10992402 33089373

[19] Diamand R Ploussard G Roumiguié M Oderda M Benamran D Fiard G. External validation of a multiparametric magnetic resonance imaging-based nomogram for the prediction of extracapsular extension and seminal vesicle invasion in prostate cancer patients undergoing radical prostatectomy. Eur Urol. (2020) 79:180–5. 10.1016/j.eururo.2020.09.037 33023770

[20] Chen T Xu L Ye L Qiu H Hu Y Liu H. A new nomogram for recurrence-free survival prediction of gastrointestinal stromal tumors: comparison with current risk classification methods. Eur J Surg Oncol. (2019) 45:1109–14. 10.1016/j.ejso.2018.12.014 30594406

[21] Vickers AJ Elkin EB. Decision curve analysis: a novel method for evaluating prediction models. Med Decis Making. (2006) 26:565–74. 10.1177/0272989X06295361 17099194 PMC2577036

[22] Vickers AJ Cronin AM Elkin EB Gonen M. Extensions to decision curve analysis, a novel method for evaluating diagnostic tests, prediction models and molecular markers. BMC Med Inform Decis Mak. (2008) 8:53. 10.1186/1472-6947-8-53 19036144 PMC2611975

[23] Mazzocca A Napolitano A. New frontiers in the medical management of gastrointestinal stromal tumours. Ther Adv Med Oncol. (2019) 11:1758835919841946. 10.1177/1758835919841946 31205499 PMC6535752

[24] Zhang H Liu Q. Prognostic indicators for gastrointestinal stromal tumors: a review. Transl Oncol. (2020) 13:100812. 10.1016/j.tranon.2020.100812 32619820 PMC7327422

[25] Lin Y Wang M Jia J Wan W Wang T Yang W. Development and validation of a prognostic nomogram to predict recurrence in high-risk gastrointestinal stromal tumour: a retrospective analysis of two independent cohorts. EBioMedicine. (2020) 60:103016. 10.1016/j.ebiom.2020.103016 32980695 PMC7522759

[26] Cao X Cui J Yu T Li Z Zhao G. Fibrinogen/albumin ratio index is an independent prognosis predictor of recurrence-free survival in patients after surgical resection of gastrointestinal stromal tumors. Front Oncol. (2020) 10:1459. 10.3389/fonc.2020.01459 33014783 PMC7462001

[27] Fletcher CD Berman JJ Corless C Gorstein F Lasota J Longley B J. Diagnosis of gastrointestinal stromal tumors: a consensus approach. Hum Pathol. (2002) 33:459–65. 10.1053/hupa.2002.123545 12094370

[28] Gold JS Gönen M Gutiérrez A Broto JM García-del-Muro X Smyrk TC. Development and validation of a prognostic nomogram for recurrence-free survival after complete surgical resection of localised primary gastrointestinal stromal tumour: a retrospective analysis. Lancet Oncol. (2009) 10:1045–52. 10.1016/S1470-2045(09)70242-6 19793678 PMC3175638

[29] Qiu Q Duan J Deng H Han Z Gu J Yue N J. Development and validation of a radiomics nomogram model for predicting postoperative recurrence in patients with esophageal squamous cell cancer who achieved pCR after neoadjuvant chemoradiotherapy followed by surgery. Front Oncol. (2020) 10:1398. 10.3389/fonc.2020.01398 32850451 PMC7431604

[30] Liu X Wu Z Lin E Li W Chen Y Sun X. Systemic prognostic score and nomogram based on inflammatory, nutritional and tumor markers predict cancer-specific survival in stage II-III gastric cancer patients with adjuvant chemotherapy. Clin Nutr. (2019) 38:1853–60. 10.1016/j.clnu.2018.07.015 30075998

[31] Liu M Song C Zhang P Fang Y Han X Li J. A nomogram for predicting cancer-specific survival of patients with gastrointestinal stromal tumors. Med Sci Monit. (2020) 26:e922378. 10.12659/MSM.922378 32449506 PMC7268888

[32] Chen Z Lin RM Bai YK. Establishment and verification of prognostic nomograms for patients with gastrointestinal stromal tumors: a SEER-based study. Biomed Res Int. (2019) 2019:8293261. 10.1155/2019/8293261 31032364 PMC6457297

[33] Joensuu H. Adjuvant treatment of GIST: patient selection and treatment strategies. Nat Rev Clin Oncol. (2012) 9:351–8. 10.1038/nrclinonc.2012.74 22525709

