# Supplementary material for: Postoperative Adjuvant Imatinib Therapy-Associated Nomogram to Predict Overall Survival of Gastrointestinal Stromal Tumor
Source: Front Med (Lausanne). 2022 Mar 10;9:777181. doi: 10.3389/fmed.2022.777181 (PMC8960199; doi:10.3389/fmed.2022.777181)
Supplement: Supplementary file 1 [file Table_1.doc]

**Table S1** Univariate and multivariate analyses of prognostic factors associated with overall survival in patients with gastrointestinal stromal tumors

|  | Univariate analysis | Multivariate analysis |  |
| --- | --- | --- | --- |
|  | HR (95 % CI) P-value | HR (95 % CI) P-value |  |
| Sex | 0.877 |  |  |
| Male | 1.00 |  |  |
| Female | 1.040 (0.634, 1.704) |  |  |
| Age (years) | 0.016 | 0.011 |  |
| <60 | 1.00 | 1.00 |  |
| ≥60 | 1.878 (1.124, 3.136) | 1.955 (1.164, 3.283) |  |
| Tumor size (cm) | <0.001 | 0.003 |  |
| ≤5 | 1.00 | 1.00 |  |
| >5 | 4.557 (2.473, 8.395) | 2.745 (1.418, 5.315) |  |
| Mitotic index (/50 HPF) | <0.001 | <0.001 |  |
| <5 | 1.00 | 1.00 |  |
| ≥5 | 4.804 (2.899, 7.963) | 3.832 (2.251, 6.525) |  |
| Tumor site | <0.001 | 0.003 |  |
| Stomach | 1.00 | 1.00 |  |
| Non-stomach | 2.839 (1.707, 4.722) | 2.244 (1.325, 3.801) |  |
| Histological subtype | 0.205 |  |  |
| Spindle type | 1.00 |  |  |
| Epithelioid/mixed type | 1.551 (0.786, 3.061) |  |  |
| Tumor rupture | 0.634 |  |  |
| No | 1.00 |  |  |
| Yes | — |  |  |
| Performance status | 0.343 |  |  |
| 0 | 1.00 |  |  |
| ≥1 | 1.271 (0.774, 2.088) |  |  |
| Diagnostic delay | <0.001 | <0.001 |  |
| No | 1.00 | 1.00 |  |
| Yes | 2.570 (1.546, 4.274) | 2.851 (1.710, 4.754) |  |
| Postoperative imatinib | 0.034 | 0.003 |  |
| No | 1.00 | 1.00 |  |
| Yes | 0.334 (0.121, 0.920) | 0.211 (0.076, 0.586) |  |
